# Reliability and Validity of the Japanese Version of the Short Form of the Expanded Version of the Posttraumatic Growth Inventory (PTGI-X-SF-J): A Cross-Sectional Study

**DOI:** 10.3390/ijerph20115965

**Published:** 2023-05-26

**Authors:** Rei Oshiro, Takafumi Soejima, Sachiko Kita, Kayla Benson, Satoshi Kibi, Koichi Hiraki, Kiyoko Kamibeppu, Kanako Taku

**Affiliations:** 1 Department of Family Nursing, Division of Health Sciences and Nursing, Graduate School of Medicine, The University of Tokyo, Tokyo 113-0033, Japan; 2 Department of Nursing, School of Nursing, Hyogo Medical University, Kobe 650-8530, Japan; 3 Department of Child Health Nursing, Graduate School of Health Sciences, Kobe University, Kobe 654-0142, Japan; 4 Department of Health Policy, National Center for Child Health and Development, Tokyo 157-8535, Japan; 5 Department of Psychology, Oakland University, Rochester, MI 48309-4482, USA; 6 Department of Social Welfare, Chinzeigakuin University, Nagasaki 854-0082, Japan; 7 Graduate Programs in Family Nursing, International University of Health and Welfare, Tokyo 107-8402, Japan

**Keywords:** posttraumatic growth, Posttraumatic Growth Inventory, PTG assessment, Japanese

## Abstract

A Japanese version of the short form of the expanded Posttraumatic Growth Inventory (PTGI-X-SF-J) was developed in this study, as the extended version captures broader, more diverse personal growth perspectives, such as existential spiritual growth. We collected cross-sectional data from 408 (first sample) and 284 (second sample) Japanese university students using the expanded version of the Posttraumatic Growth Inventory (PTGI-X-J). Exploratory factor analysis (EFA) was performed with the first sample and confirmatory factor analysis (CFA) with the second; reliability and validity were examined. The short-form version resulting from the EFA and CFA comprised 10 items and five factors. Cronbach’s alpha for the PTGI-X-SF-J total and subscale scores ranged from 0.671 to 0.875. The intraclass correlation coefficient for the total and subscale scores between the PTGI-X-J and PTGI-X-SF-J ranged from 0.699 to 0.821. Regarding external validity, no significant correlation was found between posttraumatic growth and posttraumatic stress disorder checklists. Due to its brevity, the PTGI-X-SF-J can help assess diverse spiritual and existential personal growth experiences among clients, patients, and trauma survivors while reducing physical and psychological burdens.

## 1. Introduction

Posttraumatic growth (PTG) refers to the positive psychological changes experienced due to the struggle caused by highly stressful and potentially traumatic life events [1]. Triggering events include severe life-threatening incidents and seismic experiences that have a life-changing influence [1]. Such events include natural disasters, accidents, illnesses, and relationship issues; PTG can be experienced by those who directly experience adversity, as well as those who witness it [2,3,4,5,6,7]. Many studies have used homogenous samples of people exposed to a specific type of adversity: natural disaster, serious illness, war, and violence, among others. This confines the study of PTG to a smaller sample of survivors and excludes the potential range of intentional and non-intentional adversities that people may experience in their lifetime [8]. In this context, a sample of university students can be considered as experiencing varied traumatic events. In fact, the original PTG inventory was developed using a sample of university students [9]. Since then, PTG research for university students has been conducted to develop scales and examine related factors [10,11,12,13]. Thus, university students constitute a high-functioning population that provides a representative sample of people who may be exposed to diverse intentional and unintentional adverse events.

Based on the PTG theoretical model [14], associations between PTG and other psychological variables have been reported. Posttraumatic stress disorder (PTSD) symptoms have been found to be significantly positively related to PTG [11,12]. Meanwhile, “challenge to core beliefs” has been found to be significantly positively associated with PTG [13,15]. A challenge to core beliefs is a critical factor that has been theorized to initiate the PTG process, characterized by the individual’s psychological struggle, initiated by the disruption of core beliefs that facilitates the identification of positive change and explains the variations in PTG [16]. The association between PTG and rumination has also been revealed. Two major types of rumination have been identified to understand the cognitive process that leads to PTG [17,18]). Intrusive ruminations refer to the “unsolicited invasion of one’s cognitive world; thoughts about an experience that one does not choose to bring to mind” and deliberate ruminations are constructive thoughts that are “engaged in voluntarily and can be focused purposefully on trying to understand events and their implications” [17]. PTG is significantly associated with deliberate ruminations [13,15]. Regarding the relationship between PTG and intrusive ruminations, it is somewhere between a positive relation [15] and no relation [13].

Previous studies involving people exposed to a specific type of adversity have demonstrated associations between PTG and other social and psychological variables, as the experience of traumatic life events and consequent PTG often brings about acceptance of change and reality. For example, the degree of PTG is positively associated with PTSD symptoms [19,20]. Other cross-sectional studies with adolescent earthquake survivors [21,22] and women diagnosed with infertility [23] found a positive correlation between PTG and social support. A literature review of 31 studies, including patients with breast cancer, suggested that positive cognitive coping responses, such as positive reframing, had a significant positive association with PTG [24]. Additionally, a longitudinal study among adolescents and young adults with cancer revealed that PTG could contribute to reducing psychological distress and improving quality of life (QOL) in terms of mental health [25]. PTG also contributed to improving the functional and social dimensions of QOL based on a longitudinal study conducted with liver transplant patients [26]. Moreover, PTG moderated the negative influence of PTSD symptoms on QOL among military veterans [27]. Thus, it is important for healthcare professionals to accurately assess PTG perceived by persons who directly or indirectly experience a potentially traumatic or life-altering event. Assessing PTG will be useful in developing intervention strategies to support the improvement of QOL and the well-being of these individuals from a new perspective.

In measuring PTG, the Posttraumatic Growth Inventory (PTGI) is most frequently used to assess PTG worldwide [14], for review. The original scale comprises 21 items with a five-factor structure: personal strength, new possibilities, relating to others, appreciation of life, and spiritual change [9]. Although the PTGI has been commonly used in research and clinical settings, one major concern is that this scale has many items that may cause physical and psychological burdens to people, especially those who are struggling with adversities, such as receiving aggressive medical treatments due to illness or life crises. To address this concern, the short form of the PTGI was developed by [28]. This 10-item measure comprises the same five subscales from the original inventory; each subscale has two items selected using factor loadings according to the factor structure of the original PTGI. The validity and reliability of the short form of the PTGI were tested using data from 1351 adults in the US. The short form of the PTGI has been translated into multiple languages, such as Portuguese and Spanish, but a Japanese version has not yet been developed [29,30,31], underscoring a research gap and the need to develop a short form of the PTGI for people in Japan. This tool could also advance research on PTG in Japan and the provision of clinical care for Japanese people experiencing PTG.

In 2007, the Japanese version of the PTGI (PTGI-J) was developed by Taku et al. [12], and its reliability and validity were confirmed with a sample of Japanese university students. This scale helped clarify PTG experiences among Japanese people and compare them with international samples [11,32,33]. The PTGI-J has a different factor structure than the original PTGI, with four, instead of five, factors: personal strength, new possibilities, relating to others, and spiritual change and appreciation of life (created by combining two original factors: spiritual change and appreciation of life). The four-factor structure of the PTGI-J may be due to Japanese culture and norms, especially those related to spirituality and religiosity. A previous study reported that Japanese participants tend to not evaluate some of the items, such as having a stronger religious belief, as a positive change [34]. This may be because most Japanese people do not believe in monotheism; thus, they may not share the same religious beliefs, norms, or culture as Western religious people. Another possible explanation may be the tendency of Japanese people to respect their ancestors and traditional rituals more than adhering to specific religious beliefs [34].

To resolve these culture-specific issues, and capture more diverse perspectives of personal growth, while measuring PTG with an inventory that can be applied internationally, regardless of whether religious beliefs are weak or strong, the expanded version of the Posttraumatic Growth Inventory (PTGI-X) was developed in three countries: the US, Japan, and Turkey. This version added four additional items that capture the broader contents of existential and spiritual changes [13]. These additional items of existential and spiritual change are as follows: “I have greater clarity about life’s meaning”, “I feel better able to face questions about life and death”, “I feel more connected with all of existence”, and “I have a greater sense of harmony with the world”. These items are able to measure existential connections unrelated to traditional religious beliefs that are generally experienced by individuals in cultures that are more secular (e.g., non-Western countries [13]). The validity and reliability of the PTGI-X were confirmed in the three countries mentioned above and later in 10 countries [35]. Further, it was confirmed to be useful for people with no strong religious beliefs [13]. Therefore, when developing a Japanese version of the short form of the PTGI, we considered the PTGI-X rather than the original PTGI for items concerning spirituality.

This study aimed to develop a Japanese version of the short form of the expanded version of the Posttraumatic Growth Inventory (PTGI-X-SF-J). We tested the internal consistency, corrected item-total correlations, factorial validity, concurrent validity, and external validity of the PTGI-X-SF-J. Regarding external validity, we used three variables: PTSD symptoms, challenge to core beliefs, and rumination, based on the PTG theoretical model [14] and previous research that developed PTG scales [12,13] and investigated core beliefs [15] using Japanese university students as a sample. Based on these studies’ results, we hypothesized that (a) the correlation between PTGI-X-SF-J scores and PTSD symptoms would be weakly positive, (b) the correlation between PTGI-X-SF-J scores and challenge to core beliefs would be strongly positive, (c) the correlation between PTGI-X-SF-J scores and intrusive ruminations would be weakly positive, and (d) the correlation between PTGI-X-SF-J scores and deliberate ruminations would be strongly positive.

## 2. Methods

### 2.1. Study Design

This study included two sample groups: the first and second samples comprised 408 and 284 Japanese university students, respectively. The first study was conducted from April 2016 to June 2017, and the second from September 2017 to February 2019. The analysis was conducted from February 2021 to April 2021.

### 2.2. Participants

The first cross-sectional study used questionnaires and was conducted with Japanese students at three universities in a metropolitan area of Japan. After explaining the purpose of the study to the students, those who agreed to participate and signed the consent form were asked to remain in their classroom and complete the questionnaires at their own pace. Of the 423 students who completed the questionnaires, data from 408 Japanese students (152 men, 255 women, and one other) were used for this study after excluding data from 15 participants who responded that they had never experienced any stressful event in the past.

The second cross-sectional study also used questionnaires and was conducted as part of an international joint study on traumatic exposures and PTG [35]. A total of 642 students were recruited and were requested to respond to the questionnaire, of which 371 students completed the questionnaire. Finally, data from 284 participants (96 men, 185 women, and three others) were used for this study after excluding 43 cases with missing data in 25 items of the Japanese version of the PTGI-X (PTGI-X-J) and 44 students who responded that they had never experienced any stressful event in the past.

### 2.3. Measures

#### 2.3.1. Demographics

Information on age, gender, nationality, religion, and marital status was collected from the two samples.

#### 2.3.2. Posttraumatic Growth

The PTGI-X-J [13] was used to assess PTG in both samples. This scale comprises 25 items with a five-factor structure: personal strength (four items), new possibilities (five items), relating to others (seven items), appreciation of life (three items), and existential/spiritual change (six items). Participants responded to each item using a six-point Likert scale ranging from 0 (I did not experience this change as a result of my crisis) to 5 (I experienced this change to a very great degree as a result of my crisis). Higher scores indicated greater PTG. Cronbach’s alpha in a previous study was 0.82 for personal strength, 0.84 for new possibilities, 0.87 for relating to others, 0.67 for appreciation of life, and 0.82 for existential/spiritual change [13]. The overall Cronbach’s alpha in this study was 0.922 for the first sample and 0.940 for the second sample.

#### 2.3.3. PTSD Symptoms

The PTSD Checklist for DSM-5 (PCL-5) [36,37] was used to assess PTSD symptoms in the second sample. This scale includes 20 items with a four-factor structure: intrusions (five items), avoidance (two items), negative alterations in cognitions and mood (seven items), and alterations in arousal and reactivity (six items). The participants evaluated the extent to which they were bothered by each symptom (i.e., item) using a five-point Likert scale ranging from 0 (not at all) to 4 (extremely). Higher scores indicated more PTSD symptoms. Scores ≥ 31 indicated probable PTSD [37]. Cronbach’s alpha was 0.951 for the second sample.

#### 2.3.4. Stressful Events

In the two studies, ad hoc questions were used to ask participants whether they had experienced a stressful event. The first sample inquired about 13 potential stressful events: natural disasters, accidents or injuries, serious illness, serious school or academic problems, family issues, financial or work-related issues, death of a close friend or relative, assault on you or someone you know, moving residence or changing schools, bullying or abuse, friendship problems, romantic relationship problems, and others. The second sample inquired about 19 potential stressful events: natural disasters, accidents or injuries, a serious accident at work or home, exposure to toxic substances, physical violence, violence with weapons, sexual violence, severe illness, diagnosis of cancer, serious distress, sudden artificial death of a close friend or relative, sudden accidental death of a close friend or relative, other death of a close friend or relative, being responsible for others’ accidents or injuries, domestic problems, serious school or academic problems, interpersonal relationship problems, romantic relationship problems, and others. Participants selected the most stressful event from these events and reported the degree of their perceived stress at the time it happened. Participants in the first sample responded using a seven-point scale from 1 (not at all) to 7 (extremely), and participants in the second sample responded using a five-point scale from 1 (not at all) to 5 (extremely).

#### 2.3.5. Challenge to Core Beliefs

The Japanese version of the Core Beliefs Inventory (CBI-J) [15,16] was used in the second sample to assess the degree to which one’s assumptive world was shaken or challenged by the experienced life crisis. This nine-item scale has a one-factor structure. The participants rated the degree to which they seriously re-examined their core beliefs disrupted by a specific event that they experienced on a six-point Likert scale ranging from 0 (not at all) to 5 (to a very great degree). Higher scores indicated a greater tendency to challenge one’s core beliefs. Cronbach’s alpha was 0.864 for the second sample.

#### 2.3.6. Ruminative Process

The Japanese version of the Event-Related Rumination Inventory (ERRI-J) [15,17] was used in the second study to assess intrusive and deliberate ruminations following the occurrence of the event that the participants reported. This 20-item scale has a two-factor structure. The first factor includes 10 items that capture intrusive, automatic, and undesired ruminative thoughts (ERRI-Intrusive (ERRI-I)), and the second factor includes the remaining 10 items that capture deliberate and constructive ruminative thoughts (ERRI-Deliberate (ERRI-D)). The participants rated the frequency of each rumination on a five-point Likert scale from 0 (not at all) to 4 (very often). Higher scores indicated more frequent rumination. Cronbach’s alpha was 0.955 for intrusive rumination and 0.927 for deliberate rumination in the second sample.

### 2.4. Analysis

First, descriptive statistics were obtained for the demographic variables, stress levels when a stressful event occurred, and the Japanese versions of the various scales, such as the PTGI-X, PCL-5, CBI, and ERRI. The goal was to develop a short-form version consisting of only two items associated with each of the five domains of PTG, to create a 10-item scale for ease of use, similar to the PTGI-SF. Exploratory factor analysis (EFA) of the 25 items of the PTGI-X-J was conducted using data from the first study. The number of factors was set to five because five subscales were predicted from previous studies of the development of the PTG scale [9,13,16]. EFA was conducted using the principal factor method and varimax rotation. Subsequently, a confirmatory factor analysis (CFA) of the 10 selected items was conducted using data from the second study to confirm its factor structure. The goodness of fit of the data was evaluated using degrees of freedom (χ^2^/*df*), the comparative fit index (CFI), adjusted goodness-of-fit index (AGFI), the goodness-of-fit index (GFI), and root mean square error of approximation (RMSEA [38]).

Regarding the internal consistency (i.e., reliability) of the 10 items of the PTGI-X-SF-J, Cronbach’s alphas for the total and five subscale scores were calculated using data from the second sample. Additionally, the corrected item-total correlations (CITC) for the total scores were calculated using data from the second sample. To test the concurrent validity of the PTGI-X-SF-J, intraclass correlation coefficients (ICCs) for the total score and subscale scores between the PTGI-X-J and the PTGI-X-SF-J were calculated using data from the second sample. To test the external validity of the PTGI-X-SF-J, Spearman’s rank correlation coefficients between the total and subscale scores of the PTGI-X-SF-J and the scores of the PCL-5, CBI-J, and ERRI-J were calculated. Additionally, the correlation coefficients between the total and subscale scores of the PTGI-X-J and those of the PCL-5, CBI-J, and ERRI-J were also calculated. A correlation coefficient >0.10 was considered weak, >0.30 was moderate, and >0.50 was strong [39]. IBM SPSS version 25.0 J for Windows (SPSS, Chicago, IL, USA) and IBM SPSS Amos version 25.0 (SPSS, Chicago, IL, USA) were used for statistical analyses.

### 2.5. Ethical Considerations

The study design was approved by the institutional review boards of the two affiliated universities, one being in Japan and the other in the US. Written informed consent was obtained from all study participants.

## 3. Results

### 3.1. Participants’ Demographics

The participants’ characteristics are presented in Table 1. The mean age of the first sample was 22.2 years (*SD* = 7.3); 255 participants (62.5%) were women. A total of 391 participants (95.8%) described their nationality as Japanese. Regarding the religion with which they were associated, 279 participants (68.7%) answered “non-religious”, 95 participants (23.4%) answered “Buddhism,” and eight participants (2.0%) answered “Christianity.” A total of 384 participants (94.1%) were single. The mean age of participants in the second sample was 21.2 years (*SD* = 3.2); 185 participants (65.1%) were women. A total of 281 participants (98.9%) identified their nationality as Japanese, and 282 participants (99.3%) were single.

### 3.2. Scores of the Scales

The scores of the scales are also displayed in Table 1. In the first study, the mean scores of the total PTGI-X-J and subscale scores of personal strengths, new possibilities, relating to others, appreciation of life, and existential/spiritual change were 46.0 (*SD* = 24.6), 7.7 (*SD* = 5.6), 11.4 (*SD* = 7.1), 14.0 (*SD* = 8.6), 6.4 (*SD* = 3.9), and 6.8 (*SD* = 6.0), respectively. The most common stressful events experienced by participants were serious school or academic problems (27.0%), romantic relationship problems (13.2%), and friendship problems (12.5%). The mean score of participants’ perceived stress for a selected event was 6.0 (*SD* = 1.1).

In the second study, the mean total PTGI-X-J score and subscale scores of personal strengths, new possibilities, relating to others, appreciation of life, and existential/spiritual change were 44.8 (*SD* = 27.2), 7.1 (*SD* = 5.3), 9.5 (*SD* = 6.8), 13.8 (*SD* = 8.8), 6.7 (*SD* = 4.0), and 7.8 (*SD* = 6.8), respectively. The most common stressful events experienced by participants were interpersonal relationship problems (e.g., bullying, friendships, and relationships with teachers; 19.7%), natural disasters (12.7%), and domestic problems (e.g., parental divorce and domestic violence; 12.0%). The mean score of participants’ perceived stress when the event happened was 4.0 (*SD* = 1.0). The mean total PCL score was 16.3 (*SD* = 17.4), and 58 participants (20.4%) reported scores above the cut-off value representing probable PTSD. The mean CBI, ERRI-I, and ERRI-D scores were 2.5 (*SD* = 1.2), 2.2 (*SD* = 1.2), and 1.9 (*SD* = 1.0), respectively.

### 3.3. Exploratory Factor Analysis

Of the 25 items assigned to either factor, the two items with the highest factor loadings in each factor were selected except for the fourth factor, “appreciation of life.” In the fourth factor, item 13 of the PTGI-X-J was also selected because item 23 of the PTGI-X-J was included as part of the fifth factor, representing “existential/spiritual change” in the original PTGI-X-J. Therefore, we compared the goodness of fit of the data in model 1 (with items 2 and 23 of the PTGI-X-J on “appreciation of life”) and model 2 (with items 2 and 13 of the PTGI-X-J on “appreciation of life”). The factor loadings of the selected items ranged from 0.491 to 0.813. Detailed results are shown in Table 2.

### 3.4. Confirmatory Factor Analysis

**Figure 1:**
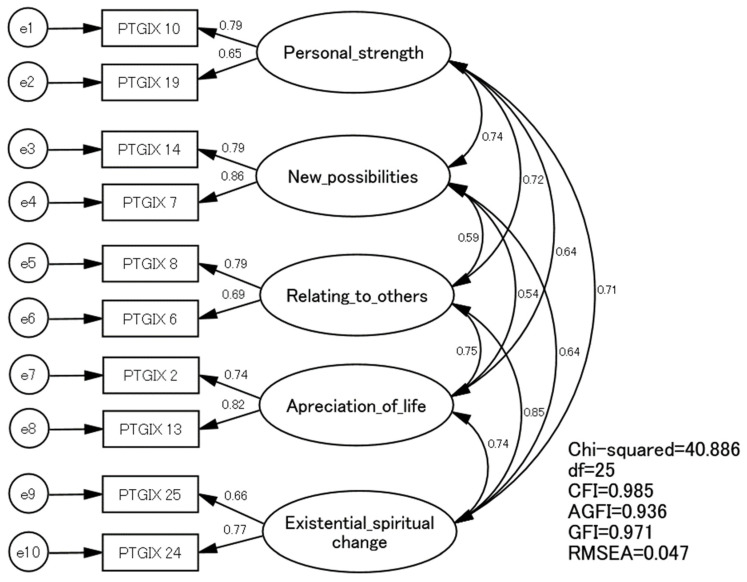
Confirmatory factor analysis using the second sample (284 Japanese students).

For the second sample, CFA was performed using models 1 and 2 selected by the EFA. After comparing the goodness-of-fit index between models 1 and 2, model 2 (with items 2 and 13 of the PTGI-X-J on “appreciation of life”) was adopted (Table 3). Model 2 with the five-factor structure using two items for each factor fit the data well: χ^2^/df = 1.64, GFI = 0.971, AGFI = 0.936, CFI = 0.985, and RMSEA = 0.047 (Figure 1). The χ^2^ score was 40.866, which was statistically significant (*p* = 0.024). The factor loadings of the 10 selected items ranged from 0.65 to 0.86.

### 3.5. Reliability

Cronbach’s alpha for the total PTGI-X-SF-J score was 0.875. For the subscales, Cronbach’s alpha was 0.678 for personal strength, 0.807 for new possibilities, 0.707 for relating to others, 0.753 for appreciation of life, and 0.671 for existential/spiritual change. The CITC between the PTGI-X-SF-J total score and the PTGI-X-J total score, excluding the 10 items of PTGI-X-SF-J, was 0.831, indicating high and positive correlations between them.

### 3.6. Validity

Regarding the concurrent validity of the PTGI-X-SF-J, the ICC for the total score between the PTGI-X-J and the PTGI-X-SF-J was 0.821. The ICCs for the subscale scores between the PTGI-X-J and the PTGI-X-SF-J were 0.863 for personal strength, 0.825 for new possibilities, 0.699 for relating to others, 0.949 for appreciation of life, and 0.728 for existential/spiritual change.

Regarding the external validity of the PTGI-X-SF-J, the variables that were significantly correlated with the total score of the PTGI-X-SF-J were the CBI and ERRI-D. The subscale score of personal strength was significantly correlated with the scores of the ERRI-D. The subscale score of new possibilities was significantly correlated with the scores of the CBI, ERRI-I, and ERRI-D. The subscale score of relating to others was significantly correlated with the scores of the CBI and ERRI-D. The subscale score of appreciation of life was significantly correlated with the scores of the CBI, ERRI-I, and ERRI-D. The subscale score of existential/spiritual change was significantly correlated with the scores of the CBI and ERRI-D. The strength of the correlation between the total and subscale scores of the PTGI-X-SF-J and CBI was almost weak, between the subscale score of the PTGI-X-SF-J and ERRI-I was weak, and between the total and subscale scores of the PTGI-X-SF-J and ERRI-D was weak to moderate. Moreover, their correlations were almost identical to those of the PTGI-X-J, except for the correlations between the subscale scores of existential/spiritual change and PCL5, appreciation of life and CBI, and existential/spiritual change and ERRI-I, as well as between the total score and ERRI-I. Detailed results are shown in Table 4.

## 4. Discussion

This study tested the factor structure, validity, and reliability of the PTGI-X-SF-J. The results demonstrated that the PTGI-X-SF-J has a five-factor structure similar to that of the PTGI-X and the original short form of the PTGI, and its model showed a good fit to the data. Additionally, regarding total score, this study confirmed moderate to good validity and reliability of the PTGI-X-SF-J using the CBI, ERRI, Cronbach’s alpha values, and CITC.

The results of factor and structure validities suggested identical conceptual structures of the PTGI-X-SF-J and PTGI-X-J. The PTGI-J has often been used in Japanese studies on PTG; however, it does not discriminate between appreciation of life and spiritual change. The PTGI-X-SF-J, however, can discriminate between the appreciation of life and spiritual change sub-concepts and measure PTG in Japanese more efficiently than the PTGI-J. Moreover, a brief measure to assess PTG reduces the physical and psychological burden of future participants and decreases the required time to complete it [40].

Although the factor structure of the PTGI-X-SF-J was the same as that of the original short form of the PTGI, the items constructed for the factors of the PTGI-X-SF-J, especially items concerning existential/spiritual change, were different from those of the original short form of the PTGI. Items for the “existential/spiritual change” factor in the PTGI-X-SF-J are “I feel more connected with all of existence” and “I have a greater sense of harmony with the world”, while items concerning the “spiritual change” factor in the original short form of the PTGI are “I have a strong religious faith” and “I have a better understanding of spiritual matters”. The newly selected two items for existential/spiritual change in the PTGI-X-SF-J were highly endorsed by Japanese participants in a previous study [34].

The current results reflect more diverse perspectives and more effectively capture the broader domain of existential and spiritual personal growth instead of narrowly focusing on religiosity. Most Japanese people do not believe in a specific religion, which was again supported by our first study, with 68.7% of participants answering that they did not believe in a specific religion. Additionally, a previous study showed that only 30% of the Japanese population have a specific religious faith, and this percentage is the lowest among developed countries [41].

Although most Japanese people do not believe in a specific religion or a higher power, they are likely to have a unique sense of spirituality and traditional spiritual behaviors in daily life [42]. This suggests that when Japanese people experience a psychological struggle due to a stressful life event, they may feel reconnected with *Kami* (gods) and spiritual forces around them. They also look for meaning in their struggle to live better lives as well as in their spiritual practices. Although these culturally unique spiritual experiences are not well captured by the original items of the PTGI-J, such as “having a stronger religious faith”, they were captured reasonably well by the items selected based on the PTGI-X-J, such as feeling “more connected with all of existence”.

Regarding the validity of the PTGI-X-SF-J, the correlations between the total and subscale scores of the PTGI-X-SF-J and the scores of the CBI and ERRI-D were significant and positive, supporting our hypotheses (b) and (d). Previous studies have indicated that persons with higher PTG are more likely to reconsider their belief systems [15,43,44,45] and think deeply, deliberately, and constructively about the meaning of the stressful events they experience [15,44,45]. Additionally, correlations between the PTGI-X-SF-J and the ERRI-I scores were not significant or had minimal values, thus partially supporting our hypothesis (c). Taku et al. [15] indicated that during a certain period after a stressful event, people are more likely to experience higher levels of intrusive ruminations (i.e., thinking unconsciously and repeatedly about stressful events) than deliberate ruminations and that this would influence their PTG. A previous study found that intrusive rumination was related to the aggravation of PTSD symptoms and depression but not to PTG, and that deliberative rumination was related to PTG [46]. These findings suggest that individuals experiencing stressful events struggle with intrusive rumination immediately after their occurrence; however, with time, deliberative rumination arises and enables individuals to seek meaning and value from the events [17], which could cause PTG. The characteristics of intrusive and deliberative rumination could thereby account for our study results. Due to the cross-sectional design of this study and the characteristics of the participants (e.g., university students of relatively younger age), the range of duration after a stressful event might be wider than that captured in this study. Additionally, because the period since a stressful event reported by each participant showed wide variations, a clear correlation between the PTGI-X-SF-J and ERRI-I was not reported in this study.

Further, while our results confirm a positive correlation regarding hypotheses (b) and (d), the strength of the correlation could not be similarly affirmed. The strength of the correlation between the total and subscale scores of the PTGI-X-SF-J and CBI was almost weak, and that between the total and subscale scores of the PTGI-X-SF-J and ERRI-D was weak to moderate. Although the previous study [15] used as a reference for the current study’s hypotheses targeted Japanese university students (similar to the sample of the present research), the event considered in that study was the Great East Japan Earthquake. By specifying the event, all participants recalled similar stress situations (e.g., tsunami images, alert sounds, and power outages) and had the same time from event to response (2 years and 3 months). Therefore, participants were likely undergoing similar psychological processes, which could be a possible reason for the weaker strength of correlation observed in this study compared to the prior research.

There was no correlation between the PTGI-X-SF-J and the PCL-5. These results did not support our hypothesis (a) or the findings of a previous study with Japanese undergraduate students [12]. Butler et al. [47] and Zebrack et al. [48] suggested that the relationship between trauma symptomatology and PTG is not linear and that it has an inverted U-shaped relationship. This suggests that having fewer PTSD symptoms is positively correlated with PTG and having more PTSD symptoms is negatively correlated with PTG. The scores of the PCL-5 in this study varied widely, which might have directly led to no linear correlations between the variables in this study. Another reason for these results may be related to the type of traumatic event experienced. The most common stressful events experienced by participants in the second sample were interpersonal relationship problems (e.g., bullying, friendships, and relationships with teachers). For university students, avoiding interpersonal relationship problems with friends, teachers, or parents is difficult. Participants who experienced these problems while they were studying, or experienced severe PTSD symptoms, may not have experienced PTG as a result of these stressful events, and if they did, it may have taken a long time to develop since these interpersonal relationships could be temporary; although, it is case-by-case. Thus, these results should be cautiously interpreted, and further studies are necessary.

The ICC of the subscale score for the factor “relating to others” was inadequate. However, from the results of the PTGI-X-SF-J and PTGI-X-J regarding external validity, this subscale is also considered as measurable as the PTGI-X-J.

### 4.1. Clinical Implications

Developing the PTGI-X-SF-J could promote the ability of healthcare professionals to assess PTG among Japanese people efficiently and with minimal burden for patients, as only 10 items are included. Furthermore, the PTGI-X-SF-J will be useful for online surveys, wherein individual attention spans tend to be shorter. Additionally, the PTGI-X-SF-J was developed using items that were more suitable for the broader spirituality of Japanese people, which enables a more accurate assessment and understanding of PTG among them. A better understanding of PTG among this group can enable the development of effective interventions that take their spirituality and existential experiences into account, thereby fostering their PTG. Finally, because the PTGI-X-SF-J was developed using the same factor structure (5 factors) and the same number of items (i.e., 10 items) as the original short form of the PTGI developed in the US, results using this scale can be compared with results of previous and future studies in other countries. Furthermore, in the German version, six out of 10 items on the PTGI-X-SF were the same as in the Japanese version in this study [40]. Not only do these PTG short scales reflect different countries and cultures, but they are also easily measurable and allow international comparisons. However, when using these PTG short scales, it is recommended to use the total score rather than each subscale score, because each subscale has only two items, and the contents of the items reflect the characteristics of each culture. If researchers want to conduct a more detailed comparison of PTG internationally, it is better to use PTGI-X. The PTGI-X-SF-J will contribute to understanding the impact of culture and its mechanisms on PTG, enabling the development of universal and culture-specific interventions to foster PTG experiences.

### 4.2. Limitations

This study had several limitations. First, the participants of this study were not representative of the general Japanese population, as they were university students. Potentially traumatic life events that our participants experienced and reported as a trigger of PTG may be different from that of other populations, such as elderly people. In addition, previous studies have suggested that college students have a higher prevalence of depression [49]; thus, this study’s results should be cautiously interpreted, and future studies should be conducted among Japanese population groups with diverse age ranges, socioeconomic backgrounds, health status, and specific traumatic experiences to confirm whether the PTGI-X-SF-J is applicable to the general Japanese population and to clarify the associations between the variables tested in this study.

Second, regarding the first sample, the number of people who were recruited is unclear. Therefore, the response rate cannot be calculated. Additionally, university students who experienced trauma to the extent that they could not answer the questionnaire were excluded from this analysis based on lack of participation in this study or lack of data; thus, caution is required in interpreting the PTG.

Third, this study used data from two cross-sectional surveys, due to which the test–retest reliability of the PTGI-X-SF-J could not be evaluated; thus, we have not been able to confirm the reliability of the PTGI-X-SF-J. To obtain evidence for the test–retest reliability of the PTGI-X-SF-J, a future longitudinal study is required.

Fourth, regarding EFA, the number of factors was set to five because five subscales were predicted from previous studies on the development of the PTG scale [9,13,16], and 10 items were selected from EFA, similar to the PTGI-SF [16]. As the final PTGI-X-SF-J excluded some items with high factor loadings on EFA, the factor structure of the PTGI-X-SF-J may become more stable if the number of items included in the short form was not limited to 10. While most of the factor loadings were above 0.40, some of the loadings on other factors were above 0.30. Although we believe that this factor loading is acceptable according to a previous study on developing the scale [50], the factor structure that includes such items may reduce its factorial validity. In addition, EFA using oblique rotations with the Kaiser’s criterion or parallel analysis should be considered in future studies, especially if researchers are interested in exploring different factor structures that better match their samples. Regarding CFA, when considering possible structures of PTG in future research, it would be useful to illustrate the applicability of several different solutions, e.g., one-dimensional, three-, four-, or five-dimensional correlation factors, second-order, or bifactor models, to further understand PTG.

Finally, there was no correlation between the PTGI-X-SF-J and PCL-5 in this study. One reason for this result is that we were unable to distinguish if the most stressful events were traumatic or not. Therefore, there is a need for a more thorough screening for PTSD with a scale measuring the degree of trauma at the time of greatest emotional damage. We believe that the relationship between trauma symptomatology and PTG is complex. To improve the accuracy of the external validity, it is necessary to review the participants and survey period for future studies carefully. Further, verifying the validity of each subscale is also needed.

## 5. Conclusions

We developed a Japanese version of the short form of the PTGI-X (PTGI-X-SF-J) to measure PTG among Japanese people and confirmed its factor structure, internal consistency, CITC, and validity. The study findings suggest that the PTGI-X-SF-J developed herein will be useful for assessing PTG efficiently and accurately, as it considers the unique spiritual beliefs and norms of Japanese people and reduces their physical and psychological burdens. Additionally, the PTGI-X-SF-J can contribute to assessing PTG in Japan, comparing PTG between countries, and developing effective and specific intervention strategies sensitive to different cultures to improve PTG research and interventions in the future. It will be useful for healthcare professionals to accurately assess PTG perceived by patients who experience a potentially traumatic or life-altering event while also supporting the improvement in patients’ QOL and well-being.

## Figures and Tables

**Table 1:** Participants’ Characteristics.

	First Sample		Second Sample	
	(*n* = 408)		(*n* = 284)	
	Mean ± *SD* ^a^ or *n* (%)			
Age (years)	22.2	±7.3	21.2	±7.3
Gender				
Male	152	(37.3)	96	(33.8)
Female	255	(62.5)	185	(65.1)
Other	1	(0.2)	3	(1.1)
Nationality				
Japan	391	(95.8)	281	(98.9)
Other	17	(4.2)	3	(1.1)
Religion				
Non-religious	279	(68.7)	-	
Buddhism	95	(23.4)	-	
Christianity	8	(2.0)	-	
Shintoism	7	(1.7)	-	
Other	17	(4.2)	-	
Marital status				
Married	21	(5.1)	2	(0.7)
Single	384	(94.1)	282	(99.3)
Other	3	(0.7)	2	(0.7)
PTGI-X-J total ^b^	46.0	±24.6	44.8	±27.2
Personal strength	7.7	±5.6	7.1	±5.3
New possibilities	11.4	±7.1	9.5	±6.8
Relating to others	14.0	±8.6	13.8	±8.8
Appreciation of life	6.4	±3.9	6.7	±4.0
Existential/spiritual change	6.8	±6.0	7.8	±6.8
PCL-5 ^c^	-		16.3	±17.4
The stress level of the event at the time ^d^	6.0	±1.1	4.0	±1.0
CBI ^e^	-		2.5	±1.2
ERRI-Intrusive ^f^	-		2.2	±1.2
ERRI-Deliberate ^f^	-		1.9	±1.0

^a^ Standard deviation. ^b^ Japanese version of the Posttraumatic Growth Inventory-Expanded. ^c^ PTSD Checklist for DSM-5. ^d^ Participants in the first sample responded using a seven-point scale (1 = not at all to 7 = extremely), and participants in the second sample responded using a five-point scale (1 = not at all to 5 = extremely). ^e^ Core Beliefs Inventory. ^f^ Event-related Rumination Inventory-Deliberate or Intrusive.

**Table 2:** Twenty-five Items of the Expanded Version of the Japanese version of the Posttraumatic Growth Inventory and Factor Loadings based on the Exploratory Factor Analysis.

Items		Factor ^a^				
		1	2	3	4	5
No. 10	I know better I can handle difficulties	0.794	0.235	0.201	0.153	0.041
No. 19	I discovered that I’m stronger than I thought I was	0.696	0.135	0.090	0.142	0.104
No. 4	I have a greater feeling of self-reliance	0.646	0.269	0.150	0.122	0.245
No. 11	I am able to do better things with my life	0.571	0.491	0.306	0.131	0.128
No. 12	I am better able to accept the way things work out	0.543	0.314	0.181	0.113	0.066
No. 14	New opportunities are available which wouldn’t have been otherwise	0.316	0.698	0.093	−0.083	0.115
No. 7	I established a new path for my life	0.374	0.686	0.187	0.023	0.179
No. 17	I am more likely to try to change things, which need changing	0.258	0.590	0.257	0.128	0.128
No. 3	I developed new interests	0.221	0.523	0.245	0.132	0.197
No. 1	I changed my priorities about what is important in life	0.147	0.357	0.177	0.265	0.111
No. 8	I have a greater sense of closeness with others	0.235	0.041	0.753	0.090	0.087
No. 6	I more clearly see that I can count on people in times of trouble	0.182	0.120	0.676	0.083	0.078
No. 21	I better accept needing others	0.089	0.242	0.661	0.206	0.081
No. 16	I put more effort into my relationships	0.049	0.389	0.559	0.219	−0.058
No. 9	I am more willing to express my emotions	0.307	0.155	0.552	0.079	0.265
No. 15	I have more compassion for others	−0.027	0.406	0.520	0.401	−0.088
No. 23	I feel better able to face questions about life and death	0.018	−0.060	0.133	0.813	0.009
No. 2	I have a greater appreciation for the value of my own life	0.093	−0.112	0.162	0.753	0.114
No. 13	I can better appreciate each day	0.195	0.291	0.304	0.541	0.104
No. 20	I learned a great deal about how wonderful people are	0.200	0.173	0.228	0.504	0.294
No. 22	I have greater clarity about life’s meaning	0.171	0.241	0.245	0.491	0.230
No. 5	I have a better understanding of spiritual matters	0.197	0.235	0.003	0.459	0.363
No. 18	I have a stronger religious faith	0.102	0.138	−0.065	0.344	0.295
No. 24	I feel more connected with all of existence	0.155	0.245	0.194	0.158	0.639
No. 25	I have a greater sense of harmony with the world	0.244	0.299	0.084	0.454	0.491

^a^ The factors are 1: personal strength; 2: new possibilities; 3: relating to others; 4: appreciation of life; 5: existential/spiritual change.

**Table 3:** Comparison of the Goodness-of-fit Index.

	χ2/df	CFI ^c^	AGFI ^d^	GFI ^e^	RMSEA ^f^
Model 1 ^a^	3.15	0.951	0.887	0.949	0.087
Model 2 ^b^	1.64	0.985	0.936	0.971	0.047

^a^ Model 1: items 2 and 23 of the PTGI-X-J were on “appreciation of life.” ^b^ Model 2: items 2 and 13 of the PTGI-X-J were on “appreciation of life.” ^c^ CFI = comparative fit index. ^d^ AFFI = adjusted goodness-of-fit index. ^e^ GFI = goodness-of-fit index. ^f^ RMSEA = root mean square error of approximation.

**Table 4:** The Correlation Coefficients Between the PTGI-X-J, PTGI-X-SF-J, and PCL-5, Experienced at the Time of the Event, CBI, and ERRI.

	PCL-5 ^c^	CBI ^d^	ERRI-I ^e^	ERRI-D ^e^
PTGI-X-J ^a^				
Total	0.058	0.360 **	0.176 **	0.456 **
Factor 1	0.023	0.239 **	0.045	0.342 **
Factor 2	0.059	0.404 **	0.192 **	0.507 **
Factor 3	0.059	0.273 *	0.211 *	0.407 **
Factor 4	−0.050	0.212 **	0.070	0.281 **
Factor 5	0.126 *	0.375 **	0.151 *	0.509 **
PTGI-X-SF-J ^b^				
Total	−0.027	0.249 **	0.086	0.369 **
Factor 1	−0.003	0.200 **	0.057	0.340 **
Factor 2	−0.005	0.372 **	0.163 **	0.432 **
Factor 3	−0.016	0.124 *	0.128 *	0.253 **
Factor 4	−0.083	0.081	−0.027	0.187 **
Factor 5	0.068	0.251 **	0.036	0.269 **

^a^ Japanese version of the expanded Posttraumatic Growth Inventory; ^b^ Japanese version of the expanded Posttraumatic Growth Inventory-Short Form; the factors are 1: personal strength; 2: new possibilities; 3: relating to others; 4: appreciation of life; 5: existential/spiritual change. ^c^ PTSD Checklist for DSM-5. ^d^ Core Beliefs Inventory. ^e^ Event-related Rumination Inventory-Intrusive or Deliberate. * *p* < 0.05, ** *p* < 0.01.

## Data Availability

The data presented in this study are available on request from the corresponding author.
